# Superior mesenteric artery aneurysm associated with chronic mesenteric ischemia in absence of identifiable risk factors and review of current literature

**DOI:** 10.1016/j.jvscit.2025.102052

**Published:** 2025-11-10

**Authors:** David A. Lieb, William Tracy, Sarvar Oreizi-Esfahani, Adana Campbell-Wright, Hruday Patel, Johnathan Allen, Tomas Heimann, Rajiv K. Chander

**Affiliations:** a Department of Surgery, Metropolitan Hospital Center, New York, NY; b Army Medical Department (AMEDD) Student Detachment, US Army Medical Center of Excellence, JBSA Fort Sam Houston, San Antonio, TX; c Department of Vascular Surgery, James J. Peters Veterans Affairs Medical Center, Bronx, NY; d Department of General Surgery, James J. Peters Veterans Affairs Medical Center, Bronx, NY; e Department of Surgery, Icahn School of Medicine at Mount Sinai, New York, NY; f Assistant Professor of Surgery at New York Medical College, Valhalla, NY

**Keywords:** Visceral artery, Aneurysm, Vein graft

## Abstract

Visceral artery aneurysms, particularly those of the superior mesenteric artery, are rare entities that can present with thrombosis and subsequent mesenteric ischemia. Most such aneurysms are classically associated with inflammatory and/or infectious etiologies, atherosclerosis, and connective tissue diseases. We present a case of an otherwise healthy 45-year-old male patient with no significant medical history or risk factors presenting with several months of abdominal pain who was subsequently found to have a symptomatic superior mesenteric artery aneurysm. Following discovery, the patient received systemic anticoagulation and later underwent open repair with an uncomplicated postoperative course.

Visceral artery aneurysms (VAAs) are rare, with a prevalence of 0.1% to 2% in the general population.^1^ Superior mesenteric artery (SMA) aneurysms account for approximately 6% of VAAs.^2^ VAAs are commonly linked to atherosclerosis and connective tissue disorders, whereas pseudoaneurysms typically arise from trauma, inflammation, or infection.^3^^,^^4^ Most VAAs are incidentally discovered but may present symptomatically, including mesenteric ischemia in SMA aneurysms.^2^^,^^3^

Given the significant risk of rupture and associated mortality, repair is generally recommended, regardless of aneurysm size. Endovascular treatment is preferred; however, open repair remains necessary for aneurysms with complex anatomy or multiple distal branches.^4^ We report a unique case of an otherwise healthy patient without risk factors or family history presenting with a symptomatic SMA aneurysm. The patient consented for the release of medical information pertinent to this report.

## Case report

The patient was a 45-year-old male with only notable medical history of asthma, who presented with a 2-week history of epigastric and left upper quadrant pain, acutely worsening over the preceding 2 days. He further reported several months of intermittent abdominal pain and nausea exacerbated by physical activity but unrelated to oral intake. He denied changes in bowel movements, prior abdominal surgery, allergies, tobacco or substance use, and had no family history of vascular or connective tissue disorders. Review of records revealed no evidence of inflammatory conditions, prior infections, or trauma aside from a remote motor vehicle collision without thoracoabdominal injury.

**Fig 1:**
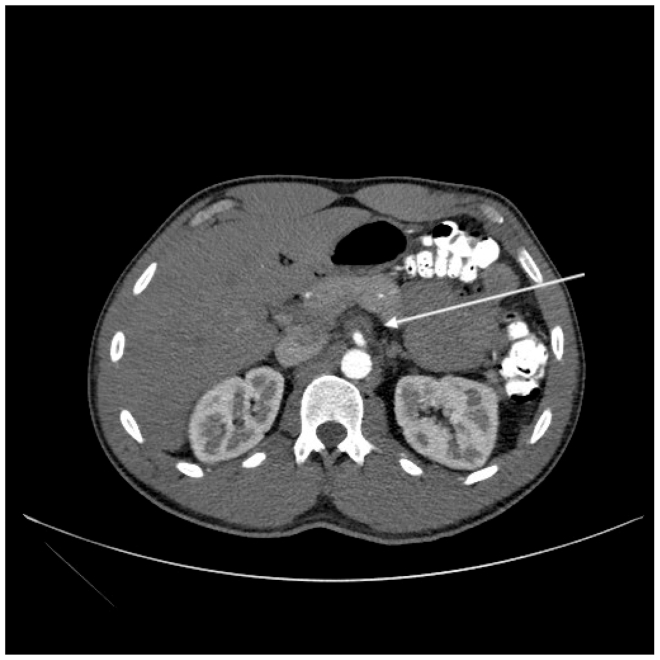
Preoperative computed tomography (CT) scan with intravenous and oral contrast highlighting superior mesenteric artery (SMA) aneurysm with large partially occlusive mural thrombus near take-off from aorta. *Arrow* highlights aneurysmal sac.

**Fig 2:**
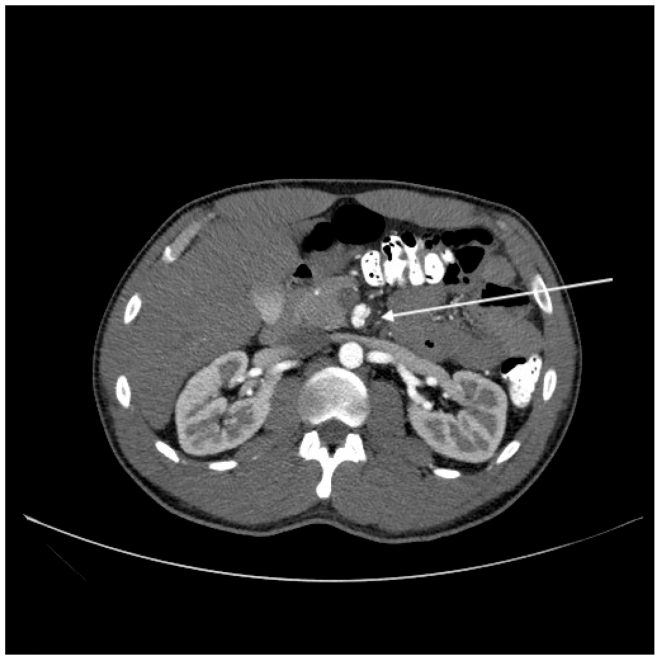
Preoperative computed tomography (CT) scan showing evidence of dissection flap approaching the take-off of the colic arteries. *Arrow* points to superior mesenteric artery (SMA) with dissection.

On presentation, vital signs were normal, and physical examination revealed only mild left upper quadrant tenderness. Laboratory studies were only remarkable for a mild leukocytosis of 13.2 × 10^9^/L (79% neutrophils). Contrast computed tomography (CT) of the abdomen and pelvis demonstrated a partially occlusive thrombus in the proximal SMA with surrounding inflammatory changes (Fig 1). The patient was admitted with a presumptive diagnosis of symptomatic SMA aneurysm, directed nothing by mouth, and started on intravenous heparin. Subsequent CT angiography (CTA) confirmed partial SMA occlusion and a 2-cm proximal aneurysm with an associated dissection flap extending into the colic branches (Fig 2).

Given the patient’s young age, colic branch involvement, and concerns regarding long-term patency of endovascular repair, we decided on open aneurysm repair with autologous greater saphenous vein (GSV). Preoperative infectious workup was negative for HIV, tuberculosis, sexually transmitted, fungal, or other significant infections.

After completion of the workup, the patient was consented for and underwent open repair via midline laparotomy. The small and large bowels were inspected with no evidence of ischemia. Following duodenal mobilization, the SMA was exposed, revealing an aneurysmal segment with dense fibrosis suggestive of chronic inflammation. Proximal and distal control were obtained, the aneurysm was opened, and mural thrombus was removed using a Fogarty balloon catheter (Supplementary Figs 1 and 2, online only). Aneurysm and thrombus cultures were sent for microbiologic and pathologic analysis. The right GSV was harvested and used for autologous interposition graft repair (Supplementary Fig 3, online only). Final inspection confirmed viable bowel, and the abdomen was closed primarily. The patient was admitted to the surgical intensive care unit for monitoring, and aside from transient pain-control issues, his postoperative course was uneventful. He was discharged on postoperative day 6 with clopidogrel 75 mg daily for a planned 90-day course.

**Fig 3:**
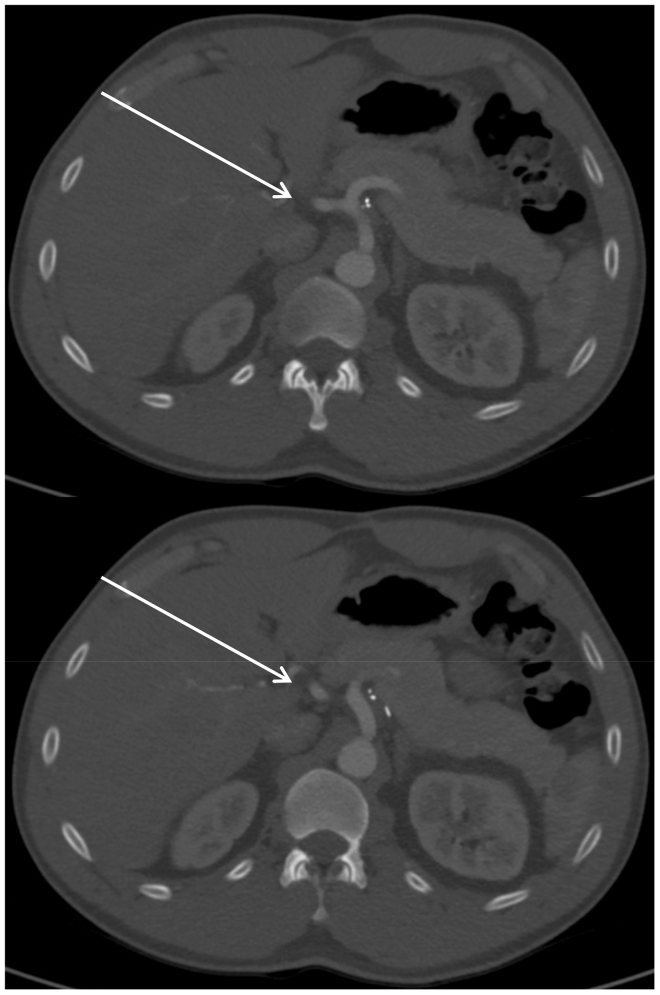
Computed tomography angiography (CTA) performed 9 months postoperatively with showing well-enhanced interposition graft and patency (*arrow*).

At 2 weeks postoperatively, the patient reported incisional discomfort but no recurrence of his prior or postprandial pain. Intraoperative cultures remained negative, and pathology returned as aneurysm likely attributed to the dissection. He gradually returned to baseline function, and by 3 months, his pain was resolved, and he had resumed normal activities, including exercise. At 9 months, he remained asymptomatic with no functional limitations, and follow-up CTA demonstrated a patent SMA and interposition graft (Fig 3).

## Discussion

VAAs are uncommon, with an estimated prevalence of 0.1% to 2%, and approximately 60% involve the splenic artery.^3^ SMA aneurysms are rarer still, accounting for about 5% of VAAs, and are typically located within 5 cm of the SMA origin, posing a risk of catastrophic mesenteric ischemia if occluded.^3^ Most VAAs are asymptomatic and incidentally detected, although some present with nonspecific or postprandial abdominal pain or rupture.^3^^,^^5^ VAAs occur most frequently in men in their sixth decade, often associated with atherosclerosis or connective tissue disease.^3^^,^^4^ Additional risk factors include inflammatory or infectious conditions, trauma, and prior surgical intervention.^5^ This case is notable for the patient’s young age and absence of traditional risk factors, underscoring the need to consider VAA in the differential diagnosis of abdominal pain, even among otherwise healthy individuals.

This carries implications for diagnosis and management. SMA aneurysms typically grow slowly, if at all, with Pitcher et al finding that, among SMA aneurysms less than 2 cm in size, none progressed to rupture and only 11% grew to larger than 2 cm.^5^ Nonetheless, Society of Vascular Surgery guidelines recommend repair of all SMA aneurysms regardless of size, as mortality due to rupture is high at 30% to 90%.^4^ As our patient presented with a symptomatic aneurysm 2 cm in size, operative repair was clearly indicated. Endovascular repair, typically involving coil embolization or covered stent placement, is typically preferred over open repair, given the reduced morbidity.^4^ However, long-term patency remains a concern compared with open repair, and certain anatomic features are not amenable to endovascular repair. In our patient, the aneurysm extended into several colic artery branches, which carried a significant risk of restenosis and precluded endovascular intervention. Therefore, we elected open repair. For open repair, the GSV, or synthetic conduit (eg, PTFE, Dacron) is commonly used.^3^ The GSV (or another native vein) is preferred in the presence of infection or a mycotic aneurysm.^6^ If the GSV is not a viable option, other graft options (such as the superficial femoral vein) and techniques have been successfully performed and described in the literature.^7^ In this case, we opted for an open repair using GSV harvest, given the ease of harvest in a healthy patient.

Although open repair is typically associated with greater postoperative complication rates and length of hospital stay vs endovascular repair, mortality is comparable, and long-term patency is typically superior. In patients with chronic mesenteric ischemia undergoing endovascular repair, 1-year patency was 83% with a 28% reintervention rate, although other studies suggest a 5-year patency of only 18%.^8^^,^^9^ Patency for open repair is significantly greater, with some studies boasting 96% to 100% patency at 5 years (although these differences were considered statistically insignificant and warrant further investigation).^8^^,^^9^ For younger patients able to tolerate surgery, we recommend open surgical repair of VAAs, rather than endovascular, given the advantage in long-term patency. Following repair, guidelines recommend annual surveillance with CTA to assess for recurrence.^4^

## Conclusions

VAAs are rare vascular entities, particularly those involving the mesenteric circulation. Although frequently asymptomatic or associated with nonspecific abdominal symptoms, their risk of rupture warrants prompt intervention. We present the case of a young, otherwise healthy patient with no identifiable risk factors who was incidentally found to have a SMA aneurysm during evaluation for vague abdominal pain. The patient underwent open repair with an uneventful postoperative recovery. This case highlights the importance of including VAA in the differential diagnosis of abdominal pain, even in younger patients without predisposing conditions. Although endovascular repair is generally first-line therapy, open surgical repair may provide superior long-term durability in select younger patients.

## Funding

None.

## Disclosures

The following manuscript represents the views of the authors alone and does not constitute an official statement or position by the Army Medical Department, the Department of the Army, the Department of Defense, the Department of Veterans' Affairs, or the US Government.
