# Supplementary material for: Superior mesenteric artery aneurysm associated with chronic mesenteric ischemia in absence of identifiable risk factors and review of current literature
Source: J Vasc Surg Cases Innov Tech. 2025 Nov 10;12(2):102052. doi: 10.1016/j.jvscit.2025.102052 (PMC12908014; doi:10.1016/j.jvscit.2025.102052)
Supplement: Supplementary Fig 1 [file mmc1.docx]

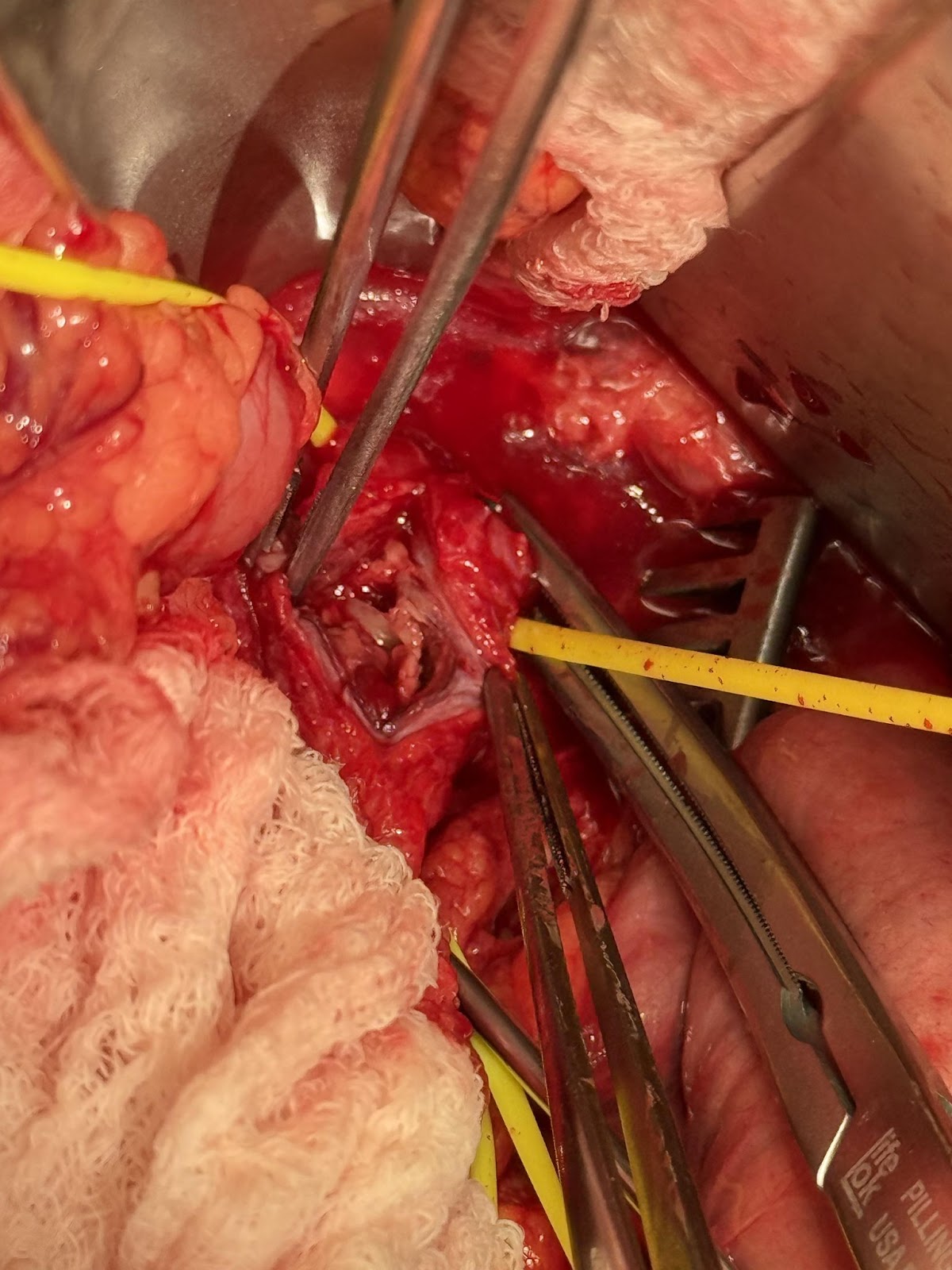


Supplemental Image 1. SMA Arteriotomy showing dissection flap involving the take-off of colic arteries and associated mural thrombus
