# Supplementary figures and images for: Superior mesenteric artery aneurysm associated with chronic mesenteric ischemia in absence of identifiable risk factors and review of current literature
Source: J Vasc Surg Cases Innov Tech. 2025 Nov 10;12(2):102052. doi: 10.1016/j.jvscit.2025.102052 (PMC12908014; doi:10.1016/j.jvscit.2025.102052)

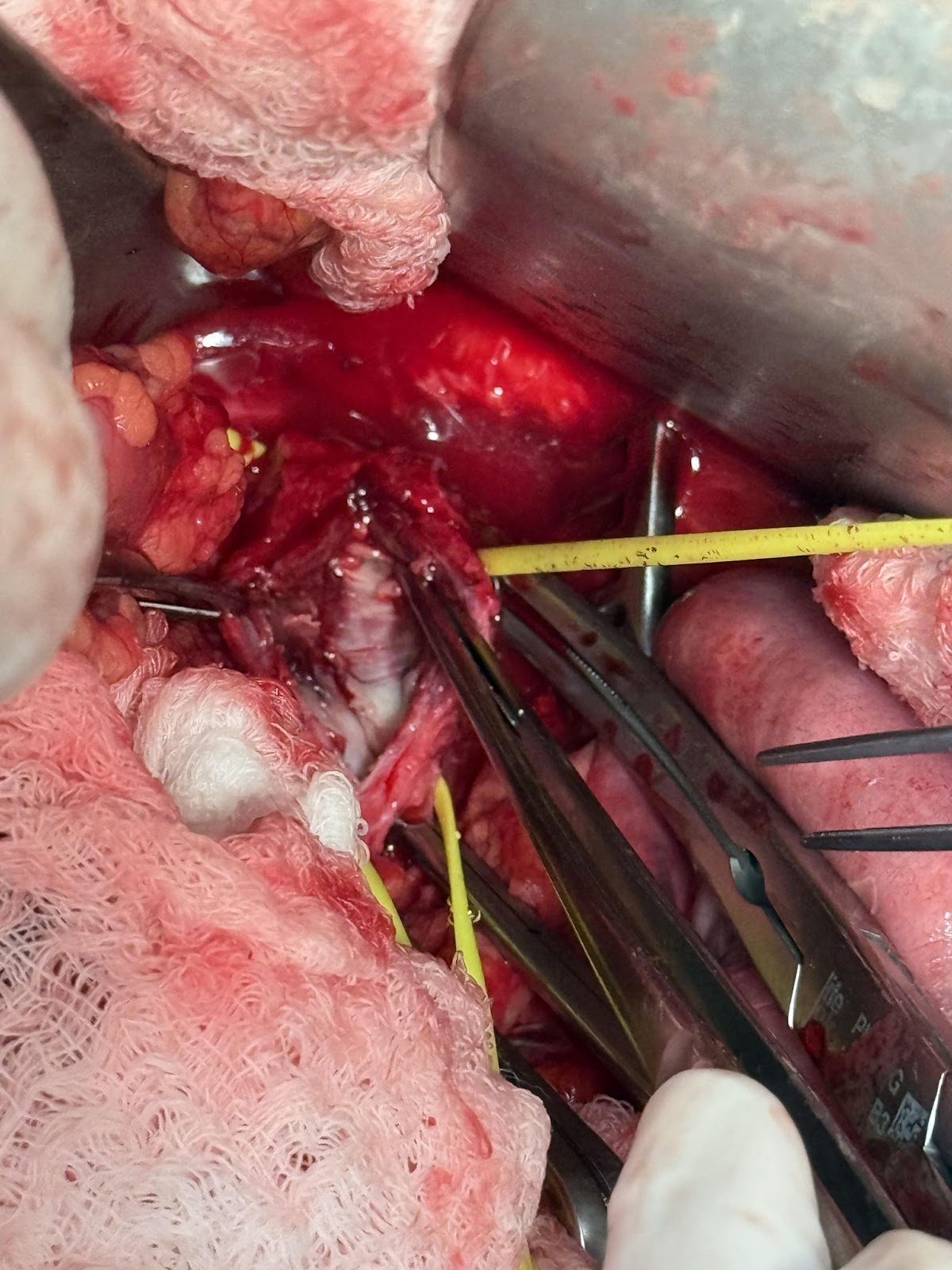


Supplemental Image 2. SMA Aneurysm sac after endarterectomy performed and removal of thrombus

Supplement: Supplementary Fig 2 [file mmc2.docx]

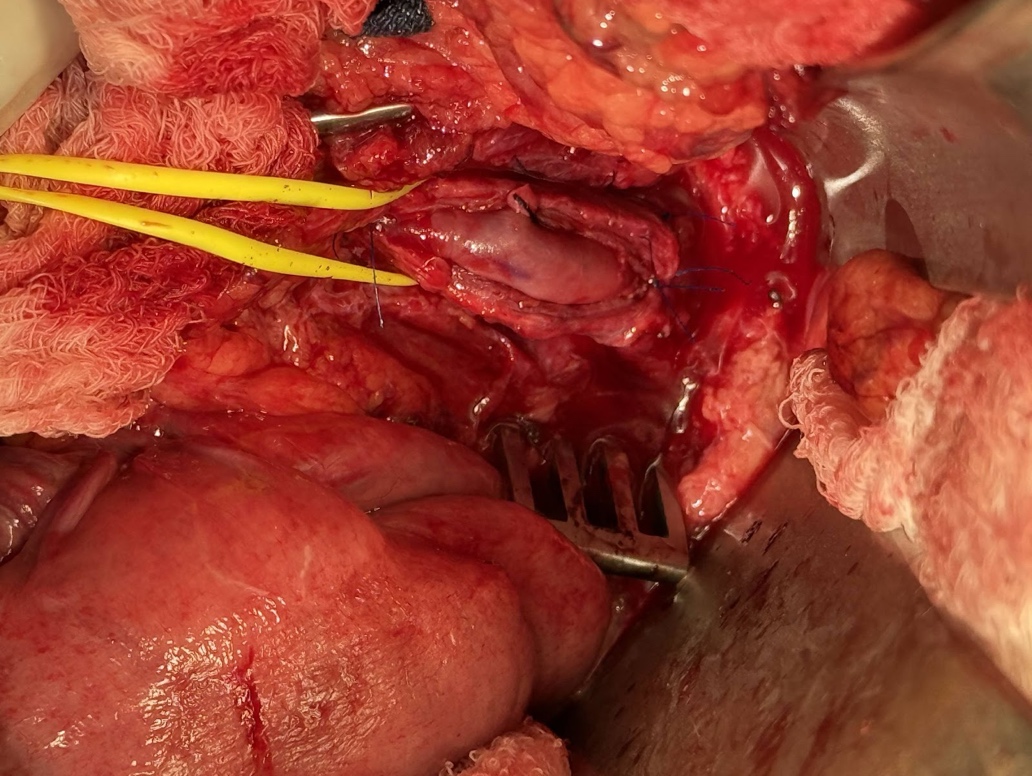


Supplemental Image 3. Proximal anastomosis of GSV to SMA

Supplement: Supplementary Fig 3 [file mmc3.docx]
